# Supplementary material for: From Stoner to local moment magnetism in atomically thin Cr2Te3
Source: Nat Commun. 2023 Sep 2;14:5340. doi: 10.1038/s41467-023-40997-1 (PMC10475109; doi:10.1038/s41467-023-40997-1)
Supplement: Supplementary file 1 — Supplementary Information [file 41467_2023_40997_MOESM1_ESM.pdf]

Supplementary Information for

From Stoner to Local Moment Magnetism in Atomically Thin Cr_2Te_3

Yong Zhong, Cheng Peng, Haili Huang, Dandan Guan, Jinwoong Hwang, Kuan H. Hsu, Yi Hu, Chunjing Jia, Brian Moritz, Donghui Lu, Jun-Sik Lee, Jin-Feng Jia, Thomas P. Devereaux, Sung-Kwan Mo & Zhi-Xun Shen

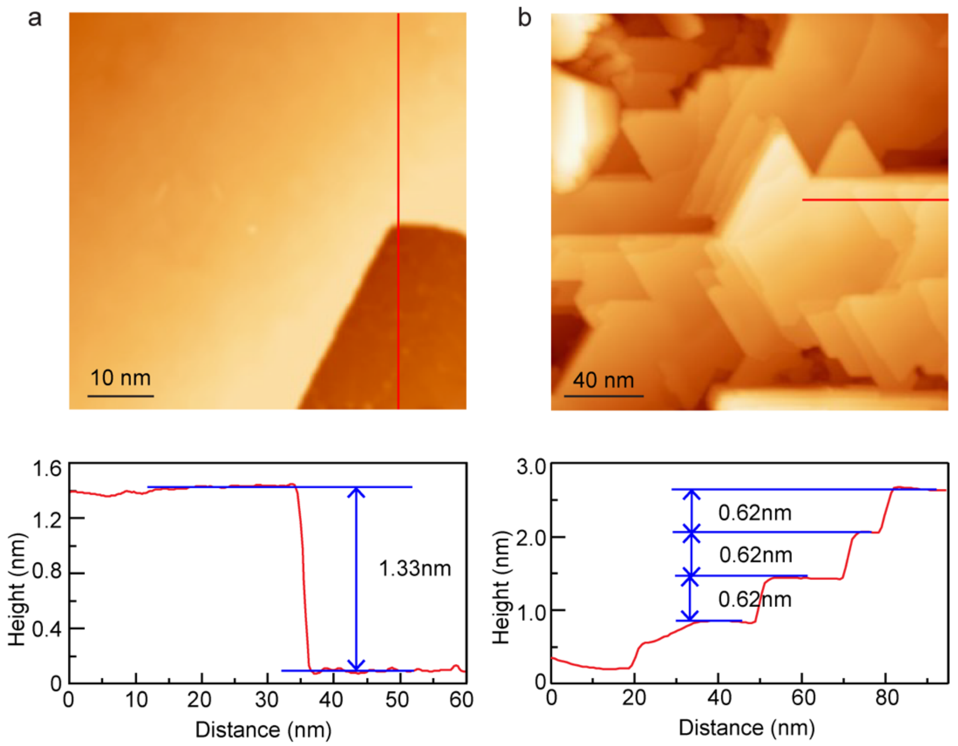

Supplementary Fig. 1 Topographic images of Cr_2Te_3 thin films on Si (111) substrate. (a) 1 ML Cr_2Te_3. The height between adjacent terraces is 1.33 nm. (b) 6 ML Cr_2Te_3. The height between adjacent terraces is 0.62 nm.

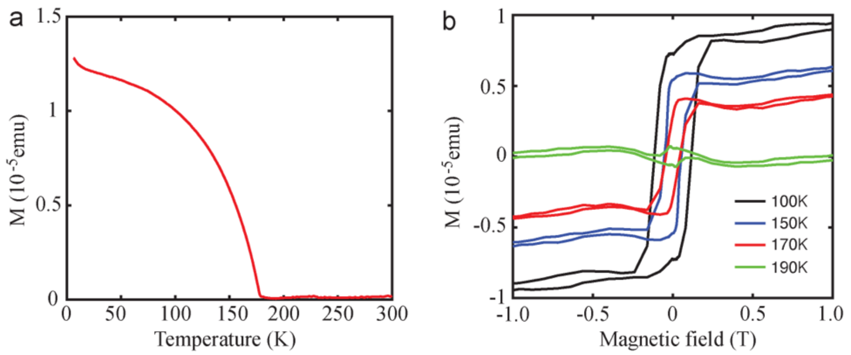

Supplementary Fig. 2 (a) Temperature-dependent magnetic moment. (b) Field-dependent isothermal magnetization.

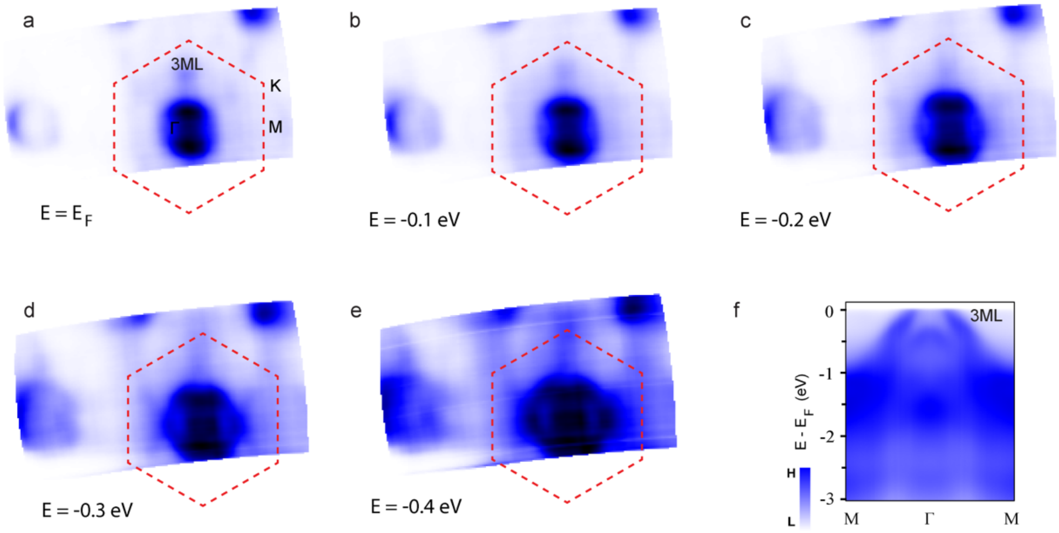

Supplementary Fig. 3 (a-e) Constant energy maps at E = E_F, -0.1eV, -0.2 eV, -0.3 eV, and -0.4 eV. Circular hole-like pockets are clearly observed around *Γ* point. (f) *s*-polarized *E*-*k* dispersions along *Γ*-M direction for 3 ML samples. The photon energy is 53 eV.

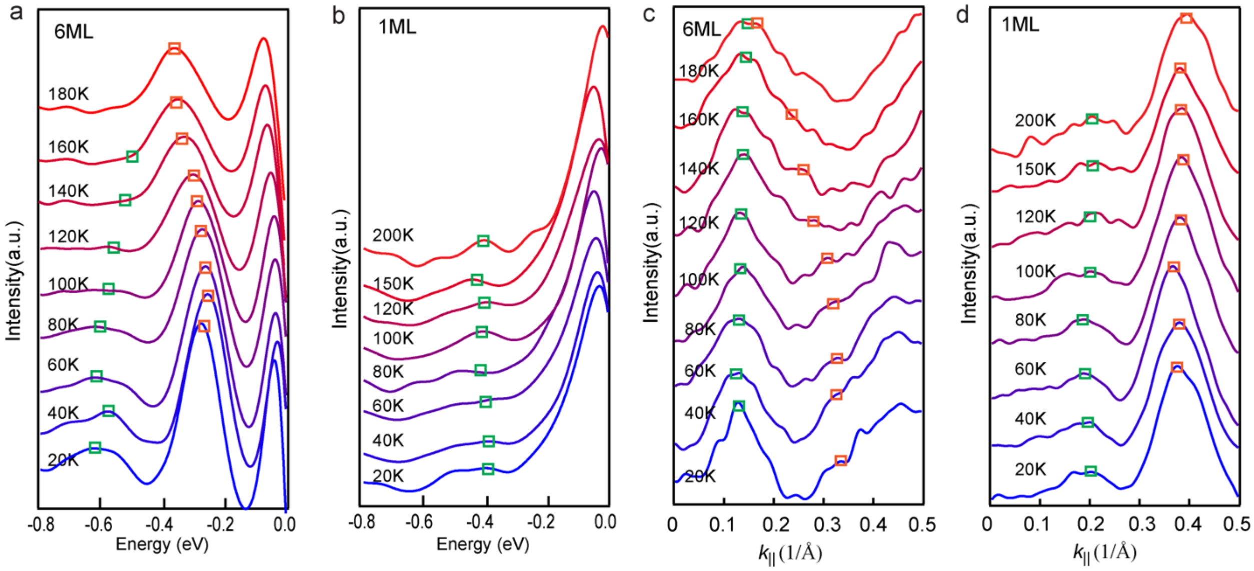

Supplementary Fig. 4 (a) T-dependent energy shift in 6 ML sample. The EDC curves are collected at momentum *k* = 0.4 (1/ Å). Green and orange squares denote the band positions in EDC curves. (b) T-dependent energy shift in 1 ML sample. The EDC curves are collected at momentum k_F. Green denotes the band positions in EDC curves. (c, d) T-dependent momentum shifts in 6ML and 1ML samples. The curves in panels c and d are collected at binding energy E_B = -0.6 eV and E_B = -0.5 eV, respectively. Green and orange squares denote the band positions in MDC curves.

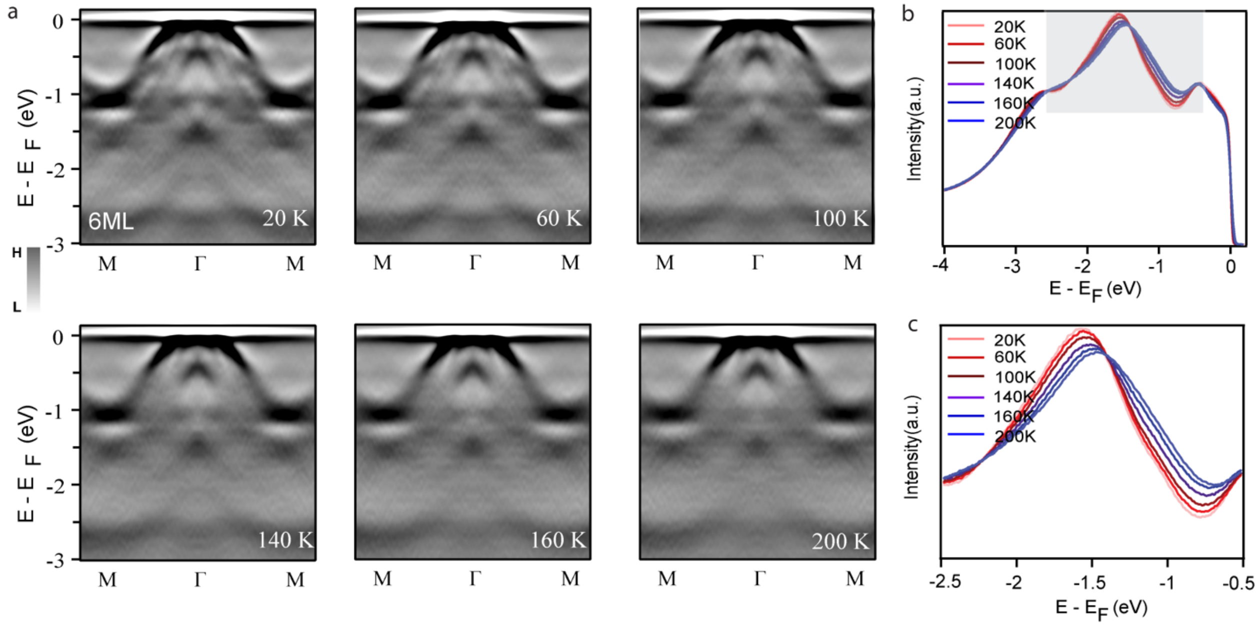

Supplementary Fig. 5 (a) T-dependent *E-k* images in 6 ML sample. All images used the second-derivative analysis method. (b) T-dependent EDC curves collected at Brillouin zone center *Γ* point. (c) Enlarged EDC curves from the gray area in panel b.

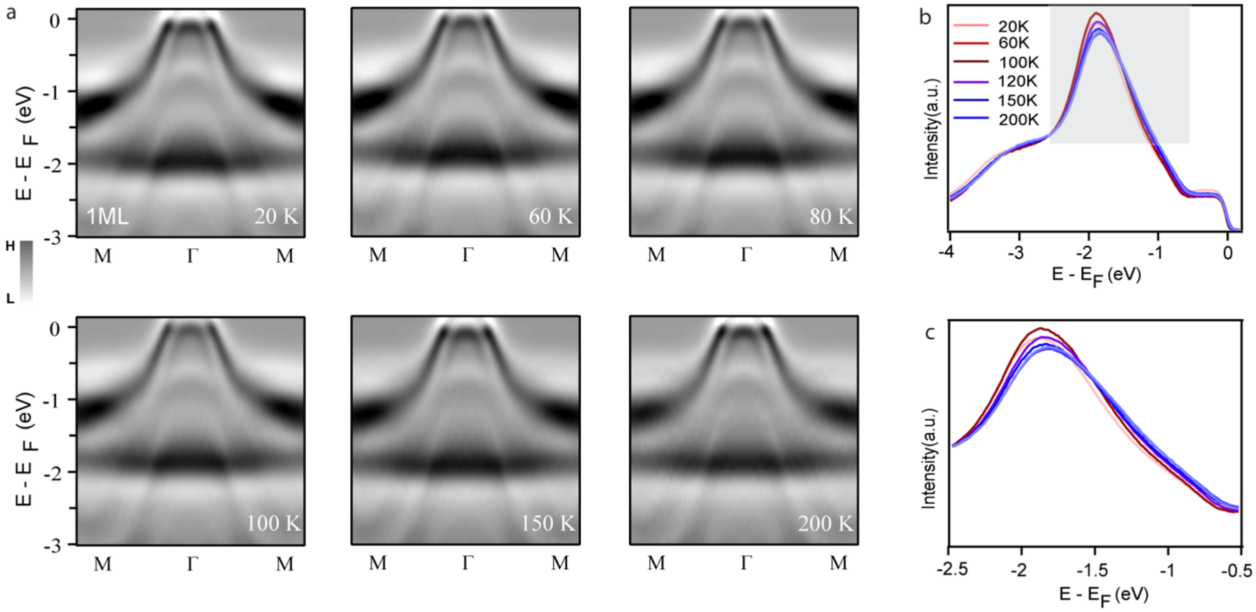

Supplementary Fig. 6 (a) T-dependent *E*-*k* images in 1 ML sample. All images used the second-derivative analysis method. (b) T-dependent EDC curves collected at Brillouin zone center *Γ* point. (c) Enlarged EDC curves from the gray area in panel b.

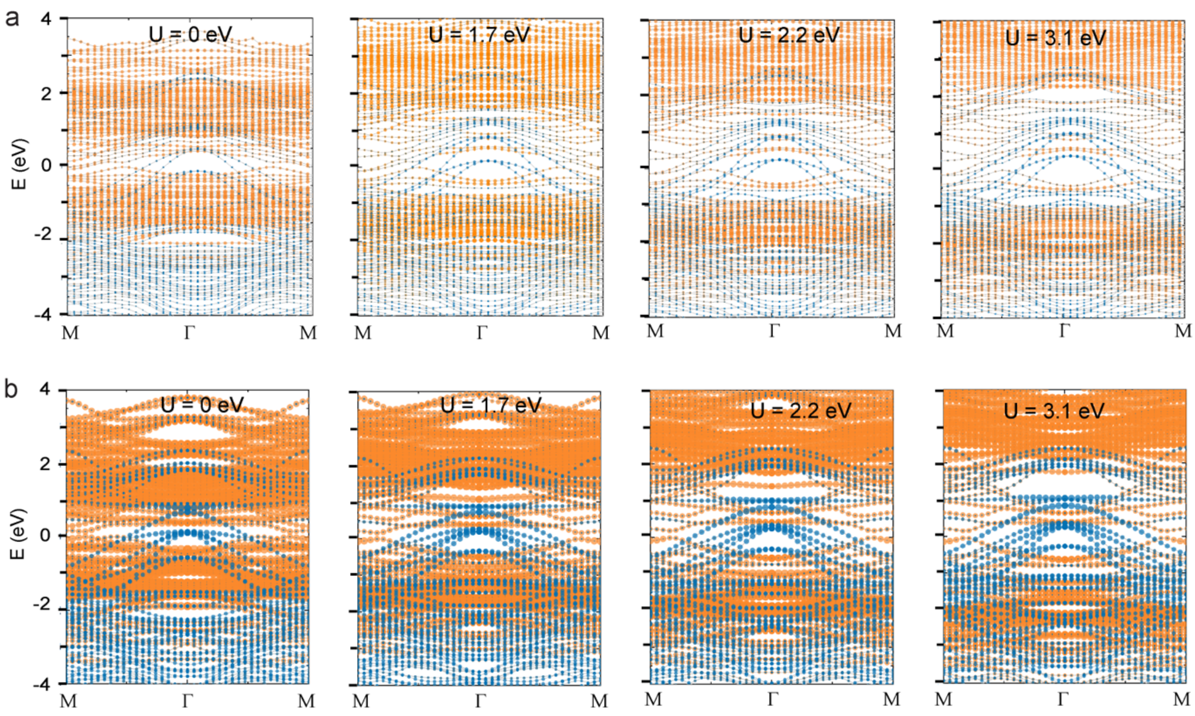

Supplementary Fig. 7 (a) DFT + U calculated band structures of 1 ML Cr_2Te_3. U = 0, 1.7, 2.2, and 3.1 eV are performed. (b) DFT + U calculated band structures of bulk Cr_2Te_3. U = 0, 1.7, 2.2, and 3.1 eV are performed.

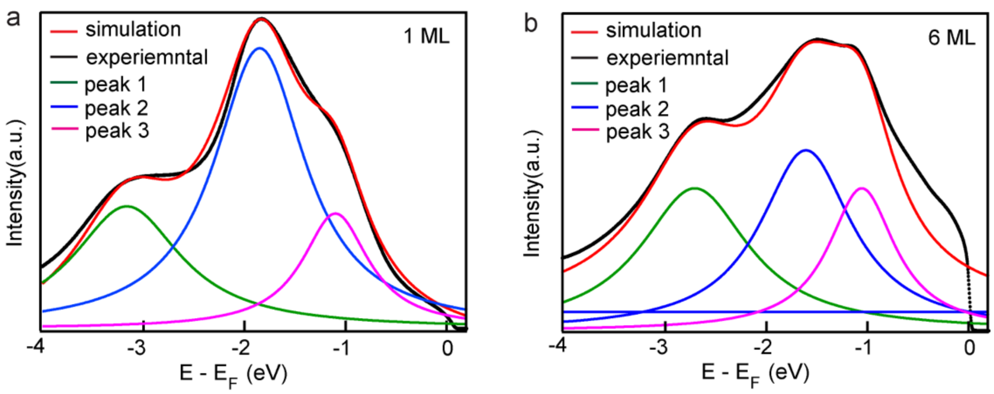

Supplementary Fig. 8 (a) Photoemission spectral analysis of 1 ML sample. Three peak positions are E = -3.16 eV, -1.85 eV, and -1.1 eV. The corresponding FWHMs are 1.3 eV, 1.1 eV, and 0.8 eV, respectively. (b) Photoemission spectral analysis of 6 ML sample. Three peak positions are E = -2.7 eV, -1.6 eV, and -1.05 eV. The corresponding FWHMs are 1.3 eV, 1.1 eV, and 0.8 eV, respectively.

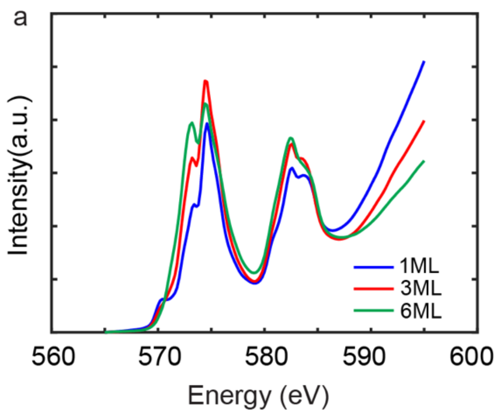

Supplementary Fig. 9 XAS spectra on 1ML, 3ML, and 6ML samples.

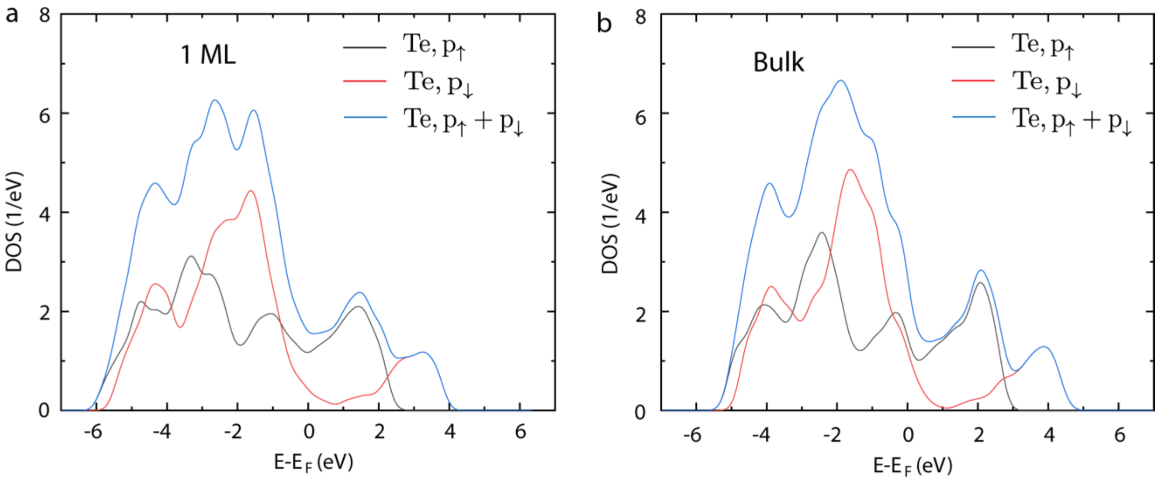

Supplementary Fig. 10 Calculated density of states of Te bands for 1 ML (a) and bulk Cr_2Te_3 (b).

Supplementary Table 1 Parameters in the Stoner model

Thickness	$D(E_F)$	$\lvert E(p\uparrow) - E(p\downarrow)\rvert$	I	$ID(E_F)$
1ML	1.61	0.18	0.45	0.72
Bulk	2.78	0.24	0.57	1.59
